# Determinants of Inequity in Health Care Services Utilization in Markazi Province of Iran

**DOI:** 10.5812/ircmj.3525

**Published:** 2013-05-05

**Authors:** Jafar Hassanzadeh, Abolfazl Mohammadbeigi, Babak Eshrati, Abbas Rezaianzadeh, Abdolreza Rajaeefard

**Affiliations:** 1 Department of Epidemiology, School of Health and Nutrition, Shiraz University of Medical Sciences, Shiraz, IR Iran; 2 Department of Epidemiology, School of Health, Qom University of Medical Sciences, Qom, IR Iran; 3 Department of Epidemiology, School of Health, Arak University of Medical Sciences, Arak, IR Iran

**Keywords:** Quality Indicators, Health Care, Utilization, Iran

## Abstract

**Background:**

National and international statistics from Iran have indicated that progresses and achievements have been made for most health indicators, but there are differences in some indicators in special groups and at the provincial level.

**Objectives:**

Our aim was to assess the main predictors of inequity in seeking Health Care Utilities (HCU) locally.

**Patients and Methods:**

Data gathered from the HCU survey, which was conducted in the Markazi province of Iran during 2008, was used in the present study. A systematic sampling method, based on the Iranian household framework, was applied in order to choose 758 households as well as 2711 individuals. The household wealth index constructed by Principle Component Analysis (PCA) and robust login link function in Generalized Estimation Equation (GEE) model were used in order to determine the predictors of inequity.

**Results:**

66.4% of those in need sought outpatient health care from which 97.7% received appropriate services. After adjustment for the clustering effect of household as well as confounding effect of the covariates, GEE model showed that there were inequities in HCU for females (OR = 2.44, CI; 1.24- 4.81) and subjects with inpatient need (OR = 2.14, CI: 1.23-3.72). Being in the lowest quintile of household wealth index was associated with the lower use of outpatient health services (OR = 0.45, CI; 0.23- 0.88).

**Conclusions:**

In spite of improvements in the national health indicator, different groups of people use health care services differently, but these inequities in HCU are related to social and individual factors. Also, it is clear that health sector facilities and the accessibility to health services are not the only predictors.

## 1. Background

Equal access for equal need is defined as equity (1). It is widely recognized that health systems are highly important determinants for improvement of the population health and performance (2). One of the most important duties of health organizations and authorities is complete coverage as well as preparation of sufficient and cost effective health care (3). In Iran, the 29th article of the constitution states that every Iranian has the right to utilize the highest attainable level of health that the Ministry of Health and Medical Education – the executor of this goal – entrusts to the medical universities at the provincial level (4). After the revolution, the coverage of social insurance was increased in Iran which resulted in an improvement in both access and accessibility to health services for over 90% of the population (5). Health care and public health services have been presented to people through a health net, which provides primary, secondary, and tertiary health services. In addition to public sectors, private sectors have a significant role in secondary and tertiary health as well as non-governmental organization work in health activities of special fields (4). National and international statistics from Iran indicated that progresses and achievements have been made in most health indicators such as the coverage of vaccination, life expectancy, infant and prenatal mortality ratios, the proportion of births attended by skilled health personnel, safe injection, combating infectious diseases such as Malaria, TB, and HIV/AIDS, as well as other indicators (3, 4, 6-10). Although public and private health sectors’ activities have improved some health indicators and despite the achievements of the Millennium Development Goals (MDGs) at the national level, there are differences at the provincial level in some indicators (11-15). Therefore, serious attempts should be taken into consideration in order to fully improve and achieve the MDG (8, 13). Health care utilization (HCU) is one of the indicators in which – despite the universal coverage and provision –inequities still exist throughout the whole country as well as among the provinces (16, 17). Based on the results of other studies, a number of predisposing factors such as sex, location of dwelling, occupation and education, enabling factors e.g. health insurance coverage, distance and availability of health care system, and factors related to the need for health care have been recognized as significant determinants of HCU (15, 18, 19). Another classification has categorized these factors into three main categories: demographic/socio-economic, personal and parental/family characteristics (18, 19). It has been assumed that these factors could create inequities in different groups of people. However, one of the most important factors, which are related to HCU is the socioeconomic position (19, 20). However, utilization of health care services can be considered as a type of individual behavior; consequently, most researchers have paid much attention to individual characteristics and have been less concerned about the impact of societal factors (21). Recently, decrease of health inequities in countries, especially in developing countries, has become a major objective for policy makers in national governments and international organizations. Determination of these disparities and differences as well as their extensiveness is a prerequisite for achieving this goal (22). In addition to inequity, monitoring and identification of health utilities and resources is necessary due to rapid increase in costs of medical care (23). Results of a study showed that there was an inequity in infant mortality at the provincial level in Iran, and that the Markazi province experienced a high inequity (24).

## 2. Objectives

The aim of this study was to assess the main socioeconomic and demographic determinants for seeking HCU in the local level rather than the entire country in order to find the main factors, which contribute to inequity.

## 3. Patients and Methods

### 3.1. Study Setting

This cross sectional study was conducted in the Markazi province, which has ten cities and is located in the center of Iran, 293 km southwest of Tehran, the capital (12). According to the latest census, 1,351,257 people including 682,367 males (50.49%) and 668,890 females (49.51%) live in this province (25). This study was conducted in all cities and a number of villages for a duration of two weeks from 16 February to 1 March 2008, based on the sampling schedule (26).

### 3.2. Subjects and Sampling

The data for the present study was gathered from the national HCU survey, which was conducted in 2008 in the Markazi province, Iran. Systematic sampling method was used to choose 760 households living in this province. The sampling method was based on the Iranian household framework available from the Health Promotion and Network Development Center of Ministry of Health and Medical Education (26). In the selected households, all members were interviewed and finally data from 2711 individuals were recorded in pre-coded and pretested questionnaires (26). In order to gather the data from subjects under 15 years old, interviewers had to contact their parents; therefore, these subjects were excluded from the study. Also written informed consent was taken from all of the subjects and the Ethics Committee in MHME approved the research protocol.

### 3.3. Instrument 

The data was collected using a valid and reliable questionnaire. This questionnaire was used for the latest HCU survey of Iran in 2002 and was revised with regards to its advantages and disadvantages. The questionnaire included some individual factors such as age, sex, marital status, job, education, socio-economic factors such as income level, and a list of assets which were used for factor analysis. In addition, seeking outpatient health services (including any type of health care such as primary health, dentistry, specialist, laboratory, et.) by people with outpatient needs within the two weeks before the interview was the outcome variable. All 15 or more years old subjects were interviewed by trained interviewers (26).

### 3.4. Statistical Analysis and Creation of the Household Wealth Index

The statistical process was done in three steps. First, we used the Principle Component Analysis (PCA) method to construct a household wealth index – based on recommendations of other studies (20, 22, 27). It was assumed that the economical status of a household reflects the socioeconomic situation of the subjects under study, thus this was the first component to be considered (20). Due to the fact that the asset data are claimed to be more reliable than other variables such as income or expenditure (27), data from the twenty five asset variables were gathered and used in the factor analysis of the present study. Number of people per number of rooms per capita, having a separate kitchen, bathroom, toilet, fully hygienic toilet, using the kitchen stove, safe heating and cooling devices, having living facilities including fridge, freezer, refrigerator, television (black and white, color, or LCD), mobile phone, clothes and dish washing machines, microwave, vacuum cleaner, computer, Internet access at home, motorcycle, car, own villa and own house were the asset variables which were included in the PCA. Scores from this primary assessment were sorted in an ascending manner. After calculating the quintiles, the lowest quintile was considered as the poorest, while the highest quintile was considered as the richest one. Second, the outcome variable was considered as a binary variable for seeking outpatient health services within two weeks prior to the interview. In this way, the percentage of the subjects who were seeking outpatient services to those who needed them was obtained. Finally, the robust logit link in the Generalized Estimation Equation (GEE) model with the exchangeable correlation matrix was used for computing the Odds Ratio (OR) as well as their 95% confidence interval (95% CI) and was applied for interpretation of the results. Robust logistic regression in the GEE model was used to adjust the clustering effect of sampling units and remove the correlation of the subjects (28, 29). The GEE model considered two sources of variances in obtaining the estimates; the interacluster and intercluster variances (30). In the GEE model, we included all socio-demographic characteristics which had a significant relationship with seeking out-patient health services in addition to those variables which had a significance probability lower than 0.35. Income level variable, however, was excluded from the model because it had a significant correlation with the household wealth index (r = 0.548, P < 0.001). We conducted all statistical analyses using the STATA statistical package (10.0). All tests were considered as two-tail and P values below 0.05 were considered as statistically significant.

## 4. Results

The response rate was 99.7%, and from all 2711 subjects who were interviewed, 2131 were 15 or older and, consequently, were included in the analysis. From the 2131 subjects, 779 needed outpatient health care. It was reported that 66.4% (517 subjects) sought outpatient health care and 97.7% (503 subjects) received it. Among the subjects who were analyzed, 59.8% were females, 45.3% lived in cities, and 73.4% were married. The mean of age and years of education of the participants was 45.33 ± 19.4 years old and 5.63 ± 5.13 years, respectively. Table 1 summarizes the findings of the bivariate analysis of socio demographic characteristics of the two group of subjects based on seeking outpatient health services. The results show that subjects that were female and had a higher household wealth index, fewer years of education, higher income level, as well as those who were insured tended to use the outpatient health services more than other subjects. Also, In-patient need was significantly related to seeking outpatient health services (P < 0.05); so that inpatient need increased outpatient utilization of health care services. However, no significant relationship was found between seeking outpatient health services and marital status, age group, supplementary insurance, insurance validity, location of dwelling, and the subjects’ job (P > 0.05). Table 2 provides the crude and the adjusted estimates of the relationship between seeking outpatient health services and the related factors. Unadjusted OR for the variables of sex, household wealth index, and inpatient need was significant in the GEE model after removing the clustering effect of the sampling method. The multivariate analysis, also showed that only three variables have significant relationships with seeking outpatient health services after adjusting the effect of other variables. According to the GEE estimates, the likelihood of seeking outpatient health services in females was 2.4 folds higher than that of males (OR = 2.44, CI; 1.24- 4.81). Inpatient need for health care services increases the likelihood of seeking outpatient health services by 2.1 folds (OR = 2.14, CI: 1.23-3.72). The relationship between seeking outpatient health services and quintile of household wealth index showed that the subjects in the first quintile had 55% lower likelihood than the subjects in the fifth quintile (OR = 0.45, CI; 0.23- 0.88).

**Table 1.** Univariate Analysis of Socio-Demographic Characteristics and Seeking out-Patient Health Services of the Subjects Under Study

Variables	Seeking Out-patient Care, No. (%)		P Value
	No	Yes	
**Sex**			0.004 ^a^
Male	39.3 (123)	60.7 (190)	
Female	29.8 (139)	70.2 (327)	
**Marital status**			0.90
Married	33.6 (192)	66.4 (380)	
Divorced and widow	35.1(27)	64.9 (50)	
Never married	33.1(43)	66.9 (87)	
**Household wealth index**			0.006 ^b^
1 St Quintile	44.4 (69)	55.8 (87)	
2nd Quintile	36.5 (57)	63.5 (99)	
3rd Quintile	31.4 (49)	68.6 (107)	
4th Quintile	30.8 (48)	69.2 (108)	
5th Quintile	25.2 (39)	74.8 (116)	
**Age Group**			0.124
15-29	28.3 (58)	71.7 (147)	
30-44	32.3 (65)	67.7 (136)	
45-59	39.6 (67)	60.4 (102)	
60 and Upper	35.3 (72)	64.7 (132)	
**The insured**			0.028 ^b^
Yes	32.3 (219)	67.7 (459)	
No	42.6 (43)	57.4 (58)	
**Supplementary Insured**			0.528
Yes	32.2 (28)	67.5 (392)	
No	67.8 (59)	32.5 (189)	
**Insurance Validity**			0.186
Yes	31.7 (200)	68.3 (430)	
No	39.5 (17)	60.5 (26)	
**Location**			0.218
City	31.4 (111)	68.6 (242)	
Rural (main)	36.5 (137)	63.5 (238)	
**Years of education**			0.028 ^b^
None (illiterature)	37.1 (95)	62.9 (161)	
1-5 (primary school)	31.1 (56)	68.9 (124)	
6-10 (guidance and high school)	38 (63)	62 (103)	
Pre-university or Diploma	20.8 (21)	72.9 (80)	
College and seminary	35.5 (27)	65.5 (49)	
**Job**			0.312
Employed	38.8 (88)	61.2 (139)	
Housewife or retired	31.5 (136)	68.5 (296)	
Student	32.8 (19)	67.2 (39)	
Unemployed	34.6 (18)	65.4 (34)	
**Income**			0.003 ^b^
Lower 200 $	39.7 (152)	60.3 (231)	
200-500 $	28 (94)	72 (242)	
Upper 500 $	29.4 (15)	70.6 (36)	
**In-patient need**			0.006 ^b^
Yes	24.7 (37)	75.3 (113)	
No	35.8(225)	64.2 (404)	

^a^Based on chi-square test

^b^Significant at the P < 0.05

**Table 2.** Crude and Adjusted or For Seeking Outpatient Health Services In GEE Model

Variables	Crude ^a^		Adjusted ^b^	
	OR (95% CI)	P value	OR (95% CI )	P value
**Gender**				
Female	1.6 (1.15- 3.17)	< 0.0001	2.44 (1.24- 4.81)	0.010
**Household wealth index**				
1 St Quintile	0.51(0.3-1.35)	0.011	0.45 (0.23- 0.88)	0.019
2nd Quintile	0.65 (0.38 -1.47)	0.101	0.68 (0.37-1.27)	0.229
3rd Quintile	0.83 (0.49- 1.63)	0.503	0.76 (0.41-1.42)	0.386
4th Quintile	0.79 (0.47-1.6)	0.382	0.77 (0.42-1.41)	0.398
5th Quintile	1		1	
**In-patient need**				
Having	2.02 (1.24-3.47)	< 0.0001	2.14 (1.23-3.72)	0.007
**Validity of insurance**				
Having	1.19 (0.6-1.82)	0.609	1.09 (0.54- 2.21)	0.809
**Place of Residency**				
City	1.23 (0.89-2.43)	0.207	1.2 (0.77-1.85)	0.423
**Years of education**				
None (illiterate)	0.97 (0.53-1.71)	0.919	1.38 (0.56-3.42)	0.491
1-5 (primary school)	1 (0.54-1.72)	0.991	1.32 (0.6 -2.92)	0.496
6-10 (guidance and high school)	0.74 (0.4-1.49)	0.332	0.98 (0.46-2.06)	0.953
Pre-university or Diploma	1.69 (0.82- 2.27)	0.155	1.71 (0.72-4.06)	0.226
College and seminary	1		1	
**Age Group, y**				
15-29	1.12 (0.71-2.03)	0.624	0.99 (0.45-2.16)	0.974
30-44	0.94 (0.59-1.81)	0.778	0.85 (0.44-1.68)	0.649
45-60	0.77 (0.48-1.61)	0.281	0.72 (.41-1.28)	0.266
Upper 60	1		1	
**Job**				
Employed	0.88 (0.43-1.54)	0.718	0.57 (0.21-1.55)	0.273
Housewife or retired	1.17 (0.59-1.81)	0.656	0.35 (0.12-1.09)	0.070
Student	0.99 (0.42-1.52)	0.975	0.50 (0.15-1.67)	0.262
Unemployed	1		1	

^a^Adjusted estimates for clustering effect of sampling method

^b^Adjusted estimates for clustering effect of sampling method and all included covariates in the GEE model

## 5. Discussion

Our results showed that 66.4% of the subjects who needed outpatient care sought it and 97.7% of them were provided with the required services. This implies that we can consider seeking of outpatient care as the HCU. Another study (19) which was conducted in Iran found these indicators to be 69.5% and 98%, respectively. Comparison of the results of the study performed in the Markazi province with those obtained from this national study showed that HCU in our study setting is the same as that of the whole country (19). The data was first analyzed in a bivariate manner; and the results showed that sex, household wealth index, insurance, education, income and in-patient need are probably factors related to the HCU. But robust logistic regression showed that only sex, household wealth index and in-patient need are the significant determinants of HCU when adjustment has been done for other covariates. These results showed that there was evidence of inequity in different groups of people; in a way that being in the lowest household wealth index and of male sex decreased the use of health care, while being in need of inpatient services increased the likelihood of seeking or utilizing health care services. Our results is the same as that of other studies which reported a significant relationship between seeking health care services and sex (31-33), household wealth index (19, 32, 33) and inpatient need (18, 33, 34). No significant relationship was observed between seeking outpatient health care services and age (18, 19, 34), education (34), insurance, location of residence (18, 34), marital status (18, 19) and job. It is significant that in the study by Hosseinpoor et al. – the only published article on utilization of health care services in Iran, conducted during 2003 – job and location of residence were significant determinants (19). These differences may be the result of a larger sample size in mobile/satellite rural people in the geographic location studied and, consequently, might have increased the power of the study for finding a significant relationship. Of course, the low coverage of insurance in rural areas during the studied year could have affected the inequity associated with geographic location. Another difference in the comparison between the study by Hosseinpoor et al. and the present research is that housewives were shown to have a significant relationship with seeking health care in the former, while sex was a significant predictor in the latter. This difference is due to the correlation between the two variables. In other words, one is the surrogate of the other. Also, it has been confirmed that the employees have less time than the unemployed for seeking health services (19). Comparisons between significant variables of bivariate and multivariate analyses informs us about the utilization of health care services. In the bivariate associations, insurance, education, and income level were considered as likely related factors. However, after adjusting the covariate variables, no significant relationship was found between seeking care services and these variables, although we had excluded income due to its significant correlation with the household wealth index. Irrespective of the household wealth index, insurance could have affected seeking health care services. However, after controlling the household wealth status, this relationship was not shown to be significant (19). Nevertheless, the relationship between education and household wealth index was clear in another study (21). Due to this correlation, years of education, also, didn’t show a significant relationship with seeking outpatient health care services in the multiple regression analysis. It has been recognized that severe diseases increase the probability of hospitalization. It seems that inpatient need can be considered as a surrogate variable for severity or comorbidity of diseases (23). Gerritsen et al. conducted a multi-ethnicity study on Afghan, Iranian, and Somali refugees and came to the conclusion that poor general health increases contact with medical specialists and utilization of health services (31). Other studies have also mentioned a relationship between the severity of disease and the need for hospitalization with seeking and utilizing health services (18, 33). Based on our results, no significant relationship was found between the place of dwelling and seeking health services, but another study which was conducted in Iran during 2003 obtained evidence of inequity in people who lived in rural areas (19). In addition to living location, the relationship between seeking care and insurance has been shown by a number of studies (19, 32, 34). This relationship was also observed in the crude analyses of the current study, but after adjusting other covariates, it was excluded from the determinant factors of the multivariate analyses. However, after the implementation of family physician program since 2005 and increasing the coverage of health insurance to over 90%, it is expected that the differences of personal density between urban and rural areas decrease and, as a result, rural people’s accessibility and utilization of health care services will increase and thus the inequity disappears (4). However, it is probable for inequity to change this pattern; it seems that access and provision of universal health insurance cannot increase the utilization of health services but could lead to decrease in catastrophic expenditures of health care. Developing countries have tried to decrease some inequities by complete coverage of insurance and other facilities for people. However, another form of inequity has been observed in these countries: higher-income groups use higher form of medical specialist services while the lower-income groups use general physicians care more (1). Although the current study showed that seeking outpatient care is related to other related factors beyond the scope of the health sector and health facilities, it has a limitation in determining the individual perception of health and health sectors. It has been mentioned that people need to pay more attention to HCU in other countries such as the USA. This attention is required because of a number of factors such as social and self-perceptions including additive consensus about the right of using health care for all, the population’s expectation of medical care, and the increased cost of medical care in private sectors (21). Therefore, investigating the influence of beliefs related to health or understanding the utilization of health services is highly suggested for further studies. In the recent decades, Iran as well as the Markazi province experienced a rapid complex pattern of mortality and morbidity. Moreover, the health system will encounter more demands in future (3, 5, 9). Therefore, the health sector needs to reform and prepare itself for new needs of the older population. The following intervention strategies are suggested for restructuring the health sector in Iran in order to achieve both equity and efficiency in health care services, these include: transition to a well-developed governance and a regulatory framework in the governance scope, increase and improvement in the delivery system of the primary health care (PHC) services and use of hospital care, pro-poor programs and interventions in order to decrease the income-related inequity for both urban and rural people, increasing the functions of quality insurance and improvement – especially for secondary level services –, and increase of insurance programs to reduce out of pocket payment (5). In addition, the recent report by the Commission on Social Determinants of Health in 2008 has advised the equity issue for all policy developments such as the HCU (35). In spite of the promotion of national health indicators and improvement in the quality and quantity of health care, there were evidences of inequity in health care utilization in different groups of people – especially males, those with low socioeconomic positions, and patients with mild diseases. Also, individual and social factors have been shown to impact health utilization and health sector facilities; and accessibility to health services is not the only predictor.
